# Adaptive Discount Factor for Deep Reinforcement Learning in Continuing Tasks with Uncertainty

**DOI:** 10.3390/s22197266

**Published:** 2022-09-25

**Authors:** MyeongSeop Kim, Jung-Su Kim, Myoung-Su Choi, Jae-Han Park

**Affiliations:** 1 Research Center for Electrical and Information Technology, Department of Electrical and Information Engineering, Seoul National University of Science and Technology, Seoul 01811, Korea; 2 Applied Robot R&D Department, Korea Institute of Industrial Technology (KITECH), Ansan 15588, Korea

**Keywords:** reinforcement learning, discount factor, uncertainty, path planning, Tetris

## Abstract

Reinforcement learning (RL) trains an agent by maximizing the sum of a discounted reward. Since the discount factor has a critical effect on the learning performance of the RL agent, it is important to choose the discount factor properly. When uncertainties are involved in the training, the learning performance with a constant discount factor can be limited. For the purpose of obtaining acceptable learning performance consistently, this paper proposes an adaptive rule for the discount factor based on the advantage function. Additionally, how to use the advantage function in both on-policy and off-policy algorithms is presented. To demonstrate the performance of the proposed adaptive rule, it is applied to PPO (Proximal Policy Optimization) for Tetris in order to validate the on-policy case, and to SAC (Soft Actor-Critic) for the motion planning of a robot manipulator to validate the off-policy case. In both cases, the proposed method results in a better or similar performance compared with cases using the best constant discount factors found by exhaustive search. Hence, the proposed adaptive discount factor automatically finds a discount factor that leads to comparable training performance, and that can be applied to representative deep reinforcement learning problems.

## 1. Introduction

Reinforcement learning (RL) is a branch of machine learning in which an agent interacts with a given environment and learns the optimal policy to achieve the predefined goal. Deep RL is a method that uses neural networks to estimate the value function or the policy in reinforcement learning. In RL, an agent makes a decision, called action, at a state according to the policy, and the action is applied to the environment. Then, the agent obtains the next state and reward from the environment. In order to compute the next action, the agent maximizes the expectation of the sum of the discounted reward signals over a finite or infinite horizon. Since the future reward is unknown, the deep RL employs neural networks to estimate the expectation, called the value function, for the future [1,2].

In real-world problems, it may be difficult to predict the value function by receiving unseen reward signals or state information, due to unexpected situations [3,4]. These problems make it difficult to estimate the value in reinforcement learning. Since the value function is computed by the reward and discount factor, it is of utmost importance to decide them properly. In particular, the focus of this paper is placed on the discount factor, which is mostly constant in the existing literature.

If the discount factor is large in an environment with high uncertainty, it may be risky to estimate the value function by considering the rewards in the distant future. Estimating the sum of rewards over a longer period of time can lead to unexpected situations due to the high uncertainty. For instance, agents can overestimate rewards in the distant future, or the convergence of the value function may not be guaranteed [5,6,7,8]. On the other hand, if the discount factor is too small, it can lead to a better generalization performance [9,10,11] but hinder the convergence speed of learning [12]. Furthermore, with the small discount factor, the reward only in the near future is taken into account in evaluating the value function, which can make short-sighted or aggressive actions. This observation motivates us to devise an adaptive rule to update the discount factor, rather than a constant.

This paper especially focuses on developing an adaptive rule for the discount factor in the policy gradient algorithms that use advantage functions [13,14]. To this end, the initial low and upper bounds of the discount factor are defined in the proposed method, and the bounds are shrunk towards a higher advantage function as the training goes on. Since it is different to computing the advantage function in on-policy and off-policy algorithms, how to apply the proposed method to both on-policy and off-policy algorithms is presented.

The discount factor by the proposed method converges to the optimal constant discount factor. Note that it is a nontrivial task to find the optimal constant discount factor when the MDP is high dimensional and the environment contains uncertainties. Moreover, the resulting training performance using the proposed method is comparable with that via the optimal constant discount factor. This means that the proposed adaptive method enables the agent to counteract the overestimation of an uncertain reward sum. To verify the performance of the proposed method, two environments are used: Tetris for the on-policy and robot motion planning for the off-policy algorithms. It is shown that the proposed adaptive discount factor outperforms the cases with constant discount factors when the environment has uncertainty. Furthermore, another adjustable algorithm in [15] gradually increases the discount factor, which means that the algorithm can get into trouble when the optimal discount factor is small. On the contrary, compared with the adjustable algorithm in [15], the proposed method can adjust the discount factor adaptively for any case, i.e., either a small or large optimal discount factor.

## 2. Related Work

Various methods are known to prevent an overestimation in reinforcement learning. Among them, the method of taking the minimum value using two or more value estimators is known to work well in various environments, and to produce good performance [5,6,16,17]. In this paper, similarly, two value estimators are used to deal with overestimation. In an environment where uncertainty exists, an agent can be trained insensitive to the uncertainty if the experience of obtaining a high reward sum is evaluated without considering uncertainty. Therefore, distributional reinforcement learning methods for estimating the distribution of the reward sum have been introduced [18,19,20]. When evaluating the value function, these methods estimate a distribution rather than a scalar value, and suppress the variance of the estimated distribution. Therefore, they do not overestimate the sum of rewards with a high-risk distribution. However, sometimes these methods may perform poorly compared to the methods for estimating the value function of a scalar value [10,21]. In this paper, we present a method to cope with uncertainty by using the discount factor so that it can be applied to the method that does not estimate the distribution.

The discount factor is a constant to reflect the value of the reward signal over time in reinforcement learning, and is generally fixed to a high value [2,21]. However, sometimes a lower discount factor leads to better learning outcomes. A high discount factor is useful if the given environment has a sparse reward problem. However, if the environment does not have the problem, a low discount factor may be considered [9]. It is also known that a low discount factor can increase the generalization performance [11,12]. However, the appropriate value for the discount factor is different depending on the environment, and several trials and errors are required to find the optimal value. To compensate for this, the discount factor can be tuned gradually from a low value to a high value [15,22]. In addition, the method of increasing the discount factor may obtain a higher performance than when a fixed discount factor is used. In this paper, similar to the above method, the discount factor is increased from a low value to a high value. At the same time, the proposed method can find an appropriate discount factor according to the given environment during the agent’s training. Additionally, we confirm that the discount factor found during training can achieve higher performance than the commonly used value.

## 3. Discount Factor in Reinforcement Learning

In this section, for the purpose of presenting the main results clearly, the reinforcement learning is reviewed and the role of the discount factor is investigated for the different environments.

### 3.1. Reinforcement Learning

The Markov Decision Process (MDP) in reinforcement learning is described by the state $s_{t}\in S$ at time step *t*, the reward $r_{t}\in R$, and the action $a_{t}\in A$. When the state $s_{t}$ is given, the agent computes the action $a_{t}$ and the computed action is applied to the environment. Afterward, the environment generates the next state $s_{t+1}$ and reward $r_{t+1}$. Then, this procedure is repeated. One iteration of the procedure is called an episode.

When the MDP is stochastic, the next state $s_{t+1}$ is determined through the state transition probability model. When the current state is $s_{t}$ and the action $a_{t}=a$ is performed, the probability of $s_{t+1}=s^{\prime }$ is defined as $T_{s,a}^{s^{\prime }}=Pr\left[s_{t+1}=s^{\prime }|s_{t}=s,\;a_{t}=a\right]$, where Pr[·] means the probability of a given event. The reward $r_{t+1}$ is determined by the predefined reward function $r(s_{t},a_{t})$, i.e., $r_{t+1}=r\left(s_{t},a_{t}\right)\in R$. The action $a_{t}$ is determined by the policy $\pi (a_{t}|s_{t})$ learned by the agent. The policy π(a|s) is the probability of the event that the action becomes *a* when the state *s* is given, i.e., $\pi \left(a|s\right)=Pr\left[a_{t}=a|s_{t}=s\right]$.

The optimal policy $\pi^{*}$ is the policy that maximizes the expected reward sum up to time $t_{end}$, and is defined as (1).

(1) $$\pi^{*}=argmax_{\pi }E_{s_{t},a_{t}}\left[\sum_{t=0}^{t_{end}}\gamma^{t-1}\cdot r\left(s_{t},a_{t}\right)\right]$$

where γ∈[0,1] means the discount factor, and E[·] means the expectation. The sum of rewards $\sum_{k=t}^{t_{end}}\gamma^{k-1}\cdot r\left(s_{k},a_{k}\right)$ is the cumulative sum of the reward signals obtained up to the end of the last episode, and is called a return $G_{t}$. The actual return can be calculated at the end of the agent’s episode. Therefore, a value function is used to estimate the sum of the discounted rewards that the agent can obtain at the present time. In RL, there are two kinds of value functions: the state value function and the action value function. The state value function $V(s_{t})$ at the state $s_{t}$ represents the expected return value obtained from the current state, i.e., $V\left(s_{t}\right)=E\left[G_{t}|s_{t}\right]$. The action value function or *Q*-function denoted by $Q(s_{t},a_{t})$ represents the expected return when the action $a_{t}$ is taken at the current state $s_{t}$. In other words, it is defined as $Q\left(s_{t},a_{t}\right)=r\left(s_{t},a_{t}\right)+\gamma V\left(s_{t+1}\right)$. The optimal state value function is denoted by $V^{*}\left(s_{t}\right)$, and the optimal action value function by *Q*-function $Q^{*}\left(s_{t},a_{t}\right)$, respectively. Rigorously, the two optimal value functions are defined as (2) and (3), respectively, according to the Bellman optimal equation.

(2) $$V^{*}\left(s\right)=\max_{a}E\left[r\left(s_{t},a_{t}\right)+\gamma V^{*}\left(s_{t+1}\right)|s_{t}=s,a_{t}=a\right]$$

(3) $$Q^{*}\left(s,a\right)=E\left[r\left(s_{t},a_{t}\right)+\gamma \max_{a^{\prime }}Q^{*}\left(s_{t+1},a^{\prime }\right)|s_{t}=s,a_{t}=a\right]$$

A method of performing a greedy policy based on the optimal value function is called value-based reinforcement learning. Greedy policy deterministically selects the action that leads to the maximum value function. In other words, the optimal policy is found from $\pi^{*}\left(s\right)={\mathrm{argmax}}_{a}Q^{*}\left(s,a\right)$. The value function is generally learned by temporal difference learning, and its value is approximated through a neural network. When the state value function is modeled through the neural network with parameter ψ and is denoted by $V_{\psi }$, the objective function $J_{V}\left(\psi \right)$ for training the neural network is given by

(4) $$J_{V}\left(\psi \right)=E_{s_{t}}\left[\frac{1}{2}{\left(r_{t}+\gamma V_{\psi }\left(s_{t+1}\right)-V_{\psi }\left(s_{t}\right)\right)}^{2}\right].$$

Temporal difference learning eliminates the need to find an actual return in learning the value function. In other words, learning can proceed without completing the episode. Most of the deep reinforcement learning methods using temporal difference learning implement an experience replay memory to store the agent’s experiences, and uses them during training through random sampling [2,16,21].

On the other hand, a method of directly learning a stochastic optimal policy is called policy-based reinforcement learning. Policy-based reinforcement learning can also model a policy through a neural network, and a policy modeled by the network parameter with parameter ϕ is denoted by $\pi_{\phi }$. The method of finding the optimal policy by tuning the parameter ϕ is called the policy gradient. The objective function $J_{\pi }\left(\phi \right)$ for training the neural network modeling the policy gradient method is given by

(5) $$J_{\pi }\left(\phi \right)=E_{\pi_{\phi }}\left[G_{t}\right]=E_{s_{t}}\left[\sum_{a_{t}}\pi_{\phi }\left(s_{t},a_{t}\right)G_{t}\right].$$

In Equation (5), the return is obtained only after the episode is finished, and the expected return is estimated through the Monte Carlo method.

Therefore, there is a disadvantage that the variance of the sampling is high and the policy cannot be learned in the middle of an episode. To alleviate this problem, the return can be estimated through a value function, and this method is called the Actor-Critic algorithm [14,23]. An actor-critic’s actor refers to the model of the policy, and the critic performs the model of the value function. The critic can be approximated by the objective function (4).

### 3.2. Role of the Discount Factor in Reinforcement Learning

In computing the return $G_{t}$, the discount factor γ plays a key role in obtaining the value function, and also the optimal policy. In order to describe the motivation of the paper in detail, this section explains how the discount factor affects the optimal policy according to the environment using numerical examples.

**Figure 1:**
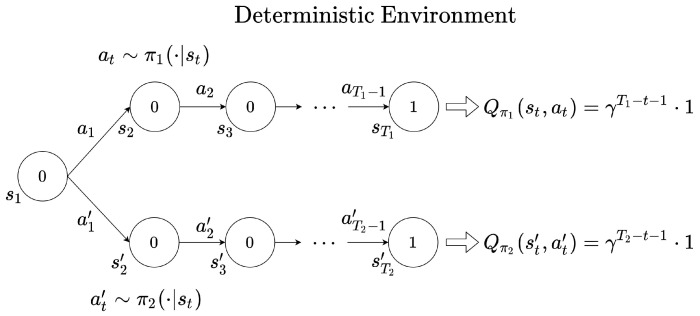
Example of trajectory in a deterministic environment.

If the discount factor is set to 1, the effect of the reward signal over time on the return is constant. Figure 1 shows how the value function is computed when a reward signal is obtained deterministically. The value inside the circle indicates the reward $r_{t}$ at the time *t*. When the reward signal $r_{T_{1}}=1$ is received at the end of the episode at time $T_{1}$, in the case of γ=1, the resulting *Q* function is 1, which is the same for any time step. That is, $Q\left(s_{t},a_{t}\right)=1\;\left(t=1,2,\cdots ,T_{1}-1\right)$. However, depending on the task goal, it may be better for the agent to obtain the reward within a faster time. Let the action value function be $Q_{\pi }$ when the policy is π. Assume that the time step $T_{1}$ is greater than $T_{2}$. If the agent follows the policy $\pi_{1}$ as in Figure 1, the agent obtains a reward at the time $T_{1}$. If the agent follows the policy $\pi_{2}$, a reward is given at $T_{2}$. Hence, if the discount factor is set to 0<γ<1, then $Q_{\pi_{1}}\left(s_{t},a_{t}\right)<Q_{\pi_{2}}\left(s_{t}^{\prime },a_{t}^{\prime }\right)$. Therefore, with a discount factor of less than 1, the closer the time point the reward signal is, the higher it is evaluated. However, if the end of the episode exists (i.e., episodic task), the optimal policy does not change according to changes of the the discount factor. As long as the discount factor is between 0 and 1, $\pi_{2}$ is always the optimal policy because $\gamma^{T_{1}-t-1}\cdot 1<\gamma^{T_{2}-t-1}\cdot 1$.

**Figure 2:**
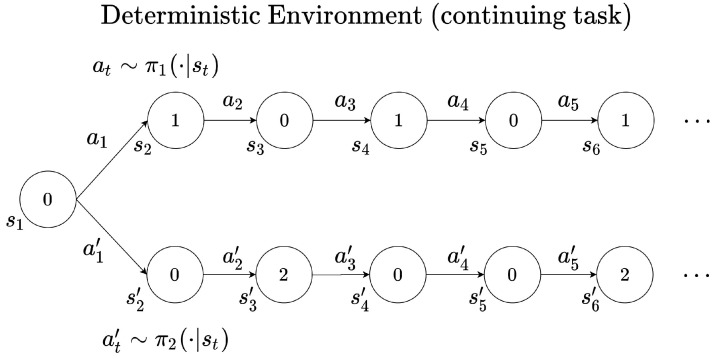
Example of trajectory in a continuing task.

On the other hand, in the case of a continuing task where the end of the episode does not exist, the optimal policy may change depending on the discount factor [11,24]. Unlike the episodic task, a continuing task is not limited to the endpoint of the episode. A continuing task does not finish the episode until the agent makes a big mistake, and the agent can receive numerous reward signals within an episode. Figure 2 illustrates an example of obtaining a reward signal from a continuing task. When the policy is $\pi_{1}$, the agent receives 1 for the reward signal every two steps. If the policy $\pi_{2}$ is followed, the agent receives 2 for the reward signal every three steps. With these policies, if γ=0.5, it follows that $Q_{\pi_{1}}>Q_{\pi_{2}}$. Hence, $\pi_{1}$ becomes the optimal policy. On the other hand, if γ=0.9, $\pi_{2}$ is the optimal policy. If the discount factor is set to 1, the maximum value function cannot be distinguished. This is because the value functions $Q_{\pi_{1}}$ or $Q_{\pi_{2}}$ are close to infinity when an agent continuously obtains reward signals. In view of these, it is observed that the optimal policy can vary according to the discount factor. In such a situation, we must redesign the reward function according to the importance of the reward signal (reward shaping). Note that rewards in the near future are more important than rewards in the distant future. By increasing the value of the reward signal in accordance with the importance, the agent may learn properly.

**Figure 3:**
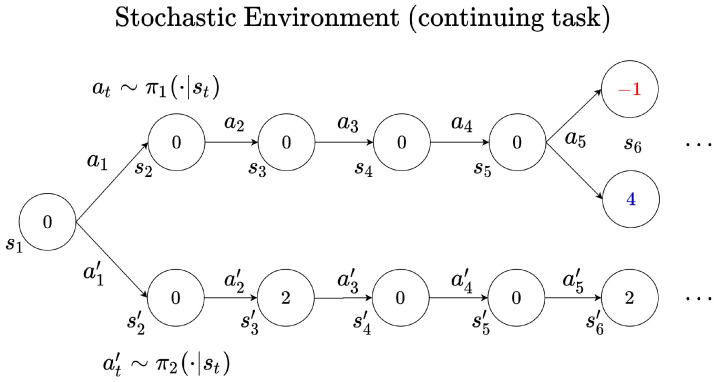
Example of trajectory in the environment with inherent uncertainty.

If there exists uncertainty in the continuing task environment, it may be difficult to learn the optimal policy with reward shaping. Figure 3 is an example of an environment with inherent uncertainty due to stochasticity. When the agent follows the policy $\pi_{1}$, it is probabilistically rewarded with $r_{6}\in \left\{-1,4\right\}$. If the agent follow the policy $\pi_{2}$, then the agent obtains the deterministic reward $r_{4}=2$. Suppose that $G_{1}$ denotes the return that the agent obtains when following the policy $\pi_{1}$ at the state $s_{1}$. Then, it can be expressed as (6) or (7).

(6) $$G_{1}=0+\gamma \cdot 0+\gamma^{2}\cdot 0+\gamma^{3}\cdot 0+\gamma^{4}\cdot 4+\gamma^{5}G_{6}$$

(7) $$G_{1}=0+\gamma \cdot 0+\gamma^{2}\cdot 0+\gamma^{3}\cdot 0+\gamma^{4}\cdot \left(-1\right)+\gamma^{5}G_{6}$$

Furthermore, at the state $s_{1}$, the state value function resulting from following the policy $\pi_{1}$ can be estimated as follows:

(8) $$V_{\pi_{1}}\left(s_{1}\right)≃0+\gamma^{4}E\left[r_{6}\right]+\gamma^{5}V_{\pi_{1}}\left(s_{6}\right)$$

In Equation (8), $E[r_{6}]$ is the average after sampling according to the distribution of the reward signal $r(s_{5},a_{5})$. The reward sum estimated by the value function $V_{\pi_{1}}$ is always different from the actual return (6) or (7) received by the agent. Accordingly, when the value function is approximated by the Equation (4), error ${\left(r\left(s_{5},a_{5}\right)-\left(V\left(s_{5}\right)-\gamma V\left(s_{6}\right)\right)\right)}^{2}$ occurs whenever the agent visits the state $s_{1}$. If such a value function is used in the policy learning, it may be difficult for the policy network to converge because of the variation in the value function induced by the error. However, if the discount factor is lowered, this error can be reduced, which means that the magnitude of $\gamma^{4}E\left[r_{6}\right]$ in the value function (8) also decreases. As a result, the overestimation of the probabilistically determined reward signal $r(s_{5},a_{5})$ is reduced as well.

**Figure 4:**
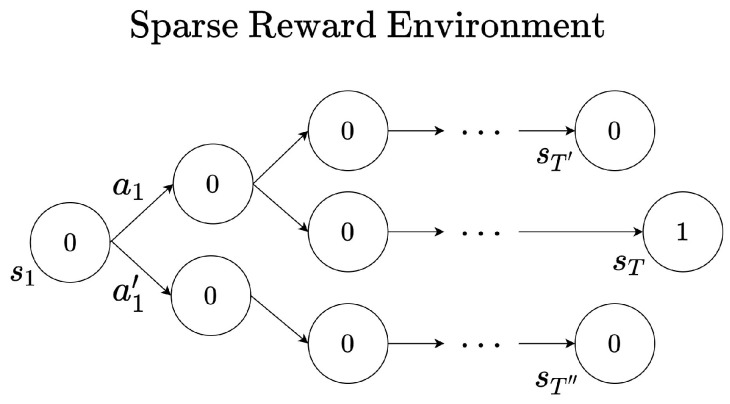
Example of trajectory in the environment with sparse reward.

There is also a possible problem when the discount factor is too low. Figure 4 describes an example of a sparse reward environment. A sparse reward environment refers to an environment in which the agent obtains meaningful reward signals once in a while. As shown in Figure 4, the reward signal is 0 in most cases. Only when the agent reaches the state $s_{T}$ does it obtains a meaningful reward signal $r_{T}=1$. It is not easy for a less trained agent to find these reward signals while interacting with the environment. Even if the reward signal is found by the agent, if the reward sum is estimated through the value function, it may be difficult to predict the value at a state far away from the reward signal. The *Q* function $Q(s_{1},a_{1})$ at state $s_{1}$ can be estimated as $Q\left(s_{1},a_{1}\right)=\gamma^{T-1}\cdot 1$ after obtaining reward $r_{T}\;=\;1$. This estimated reward sum can be evaluated to be lower when the time step *T* is large or the discount factor is low. Therefore, since this makes only very little difference between $Q(s_{1},a_{1})$ and $Q(s_{1},a_{1}^{\prime })$, the agent can not be sure that $a_{1}$ is indeed a rewarding action.

As explained earlier, the discount factor has a great influence on learning the optimal policy according to the given environment. A high discount factor can be advantageous for learning when the reward signals are sparse. However, if there is uncertainty in the environment, a high discount factor may rather hinder the convergence of learning. Therefore, depending on the given environment, there must be the most appropriate discount factor for learning. With this observation in mind, in this paper, an adaptive discount factor method is proposed, such that it can find an appropriate value for the discount factor during learning.

## 4. Adaptive Discount Factor in Reinforcement Learning

### 4.1. Adaptive Discount Factor

The discount factor has to be adjusted in consideration of environmental uncertainty and the sparsity of the reward [9,11,12]. Without these considerations, the agent can show lower performance. To this end, we want to define an evaluation function of an agent’s performance according to the current discount factor. Additionally, based on the evaluation function, we want to find an update rule for the discount factor that can improve the agent’s performance.

The definition of the return with a specific discount factor $\gamma_{1}$ is defined by the Equation (9). The return $G_{t}\left(\gamma_{1}\right)$ is the sum of rewards obtained by the current policy learned using $\gamma_{1}$. However, if the future reward signal is obtained probabilistically or if there is a risk (probabilistically negative reward) in the future reward signal, then the return can be overestimated.

(9) $$G_{t}\left(\gamma_{1}\right)=r_{t+1}+\gamma_{1}\cdot r_{t+2}+\gamma_{1}^{2}\cdot r_{t+3}+\cdots +\gamma_{1}^{T-1-t}\cdot r_{T}$$

A high discount factor can lead to an overestimation for the reward in the future when there is uncertainty or stochasticity. By considering uncertainty or risk in the environment and reward overestimation due to an inappropriate discount factor, the agent has to be able to measure confidence for the reward or return. If the expectation of the return is known, it is possible to estimate the sum of the return that is probabilistically determined. For instance, suppose that the agent obtains the sum of the reward $G(\gamma_{1})$ generated by the current policy and the discount factor $\gamma_{1}$. If the sum of the reward (i.e., $G_{t}\left(\gamma_{1}\right)$) is larger than its expectation (i.e., $E[G_{t}\left(\gamma_{1}\right)]$), then the positive future rewards for the current policy is guaranteed. In this case, the high discount factor does not make any overestimation and it can be increased. On the other hand, if the return yielded by the current policy is less than or equal to the expected return, the large reward sum in the future is not guaranteed or a lower reward sum can be made. For this case, since the high discount factor can result in overestimation, the discount factor needs to be decreased. To this end, the return confidence corresponding to the current policy is measured by $\frac{1}{M}\sum^{M}\left(G_{t}-E\left[G_{t}\right]\right)$, where *M* denotes the number of data points. However, the expected return does not know its exact value during training. Hence, the expected return can be replaced by the corresponding value function as follows:

(10) $$\frac{1}{M}\sum^{M}\left(G_{t}-E\left[G_{t}\right]\right)≃\frac{1}{M}\sum^{M}\left(G_{t}-V\left(s_{t}\right)\right),$$

where the right hand side of this equation is called an advantage function and is denoted by $A_{t}$. The advantage function can be used to evaluate the trained policy using the discount factor. Similarly, the advantage function can also be expressed as

(11) $$A_{t}=\frac{1}{M}\sum^{M}\left(Q\left(s_{t},a_{t}\right)-V\left(s_{t}\right)\right).$$

The proposed adaptive discount factor algorithm is devised by evaluating the performance of the current discount factor using the advantage function. When the advantage function is low, it is interpreted that the discount factor can lead to overestimation. Hence, the discount factor needs to be decreased. Conversely, if the advantage function is high, it means that the current policy is learning via a high reward sum, which suggests that the discount factor should be increased.

The proposed adaptive discount factor is assumed to be used during only the transient time in training. This is because it is also observed that a constant discount factor shows acceptable performance after the transient period of the training.

The proposed adaptive algorithm has mainly two tuning parameters: $\gamma_{1}$ and $\gamma_{2}$. $\gamma_{1}$ is used as the discount factor for the current policy, its initial condition is 0.5, and the algorithm increases $\gamma_{1}$ to obtain a higher advantage function. $\gamma_{2}$ is used as the baseline and the upper bound of $\gamma_{1}$. The initial condition of $\gamma_{2}$ is 0.99. Depending on the advantage function, the proposed adaptive algorithm decreases $\gamma_{2}$.

Let $A_{t}\left(\gamma \right)$ denote the advantage function with the discount factor γ and time *t*. The proposed adaptive algorithm adjusts the discount factor $\gamma_{1}$ and $\gamma_{2}$, such that it results in the highest advantage function between the upper and lower limits, and is summarized as

(12) $$\left(\gamma_{1},\gamma_{2}\right)=\left\{\begin{array}{cc} \gamma_{1}\leftarrow \gamma_{1}+c\cdot \left(1/\sigma_{t}\right)\; & \mathrm{if}\;A_{t}\left(\gamma_{1}\right)<A_{t}\left(\gamma_{2}\right)\\ \gamma_{2}\leftarrow \gamma_{2}-c\cdot \left(1/\sigma_{t}\right)\; & \mathrm{else}\;\mathrm{if}\;A_{t}\left(\gamma_{1}\right)>A_{t}\left(\gamma_{2}\right)\\ \mathrm{do}\;\mathrm{nothing}\; & \mathrm{else}\;\mathrm{if}\;\gamma_{1}=\gamma_{2} \end{array}\right.$$

where $\sigma_{t}$ is the correction function defined as

(13) $$\sigma_{t}=1+\frac{|\delta_{t-1}-\delta_{t}|}{\delta_{t}},$$

$\delta_{t}={\left(r_{t}+\gamma V_{\psi }\left(s_{t+1}\right)-V_{\psi }\left(s_{t}\right)\right)}^{2}$, and the parameter *c* is the stepsize. The first line of (12) means that $\gamma_{1}$ is increased when $A_{t}\left(\gamma_{1}\right)$ is smaller than the baseline advantage function $A_{t}\left(\gamma_{2}\right)$. On the other hand, the second line of (12) implies that $\gamma_{2}$ is decreased when the baseline advantage function $A_{t}\left(\gamma_{2}\right)$ is smaller than $A_{t}\left(\gamma_{1}\right)$. Note that $A_{t}\left(\gamma_{2}\right)<A_{t}\left(\gamma_{1}\right)$ can imply that the baseline is not good enough, and hence, the baseline needs to be decreased. By doing this, the baseline can be improved, which can lead to a better discount factor $\gamma_{1}$ afterward. In view of the definition of $\delta_{t}$, naturally, it is large at the beginning of training and its variation becomes smaller as the training goes on. The rationale behind the definition of $\sigma_{t}$ is that the adaptation rule changes the discount factor during the transient period of the training since the value function is not reliable in the beginning.

According to the rule of the adaptive discount factor described in (12), the following holds.

(14) $$A_{t}\left(\gamma_{1,k}\right)\leq A_{t}\left(\gamma_{1,N}\right),$$

where *k* is the number of training epochs and *N* is the terminal epoch. Hence, $\gamma_{1,k+1}=\gamma_{1,k}+c\cdot \left(1/\sigma_{t}\right)$ when $A_{t}\left(\gamma_{1}\right)<A_{t}\left(\gamma_{2}\right)$. The discount factor $\gamma_{1}$ for training the current policy is updated according to $\gamma_{2}$ and its advantage function. This is because $\gamma_{1,k}\leq \gamma_{1,k+1}\leq \gamma_{2}$, $\gamma_{1,k}$ is always less than or equal to $\gamma_{1,k+1}$ because of their update rule (12). Note that 0<γ<1, $\gamma_{1}$ is always non-decreasing, and $\gamma_{2}$ is always non-increasing. According to the definition of return (9), (14) can be written as

(15) $$\begin{array}{cc} \; & \sum_{l=t}^{t_{end}}\gamma_{1,k}^{l-1}\cdot r_{l}-E\left[G_{t}\left(\gamma_{1,k}\right)\right]\\ \; & \;\;\;\;\;\leq \sum_{l=t}^{t_{end}}\gamma_{1,N}^{l-1}\cdot r_{l}-E\left[G_{t}\left(\gamma_{1,N}\right)\right]. \end{array}$$

**Figure 5:**
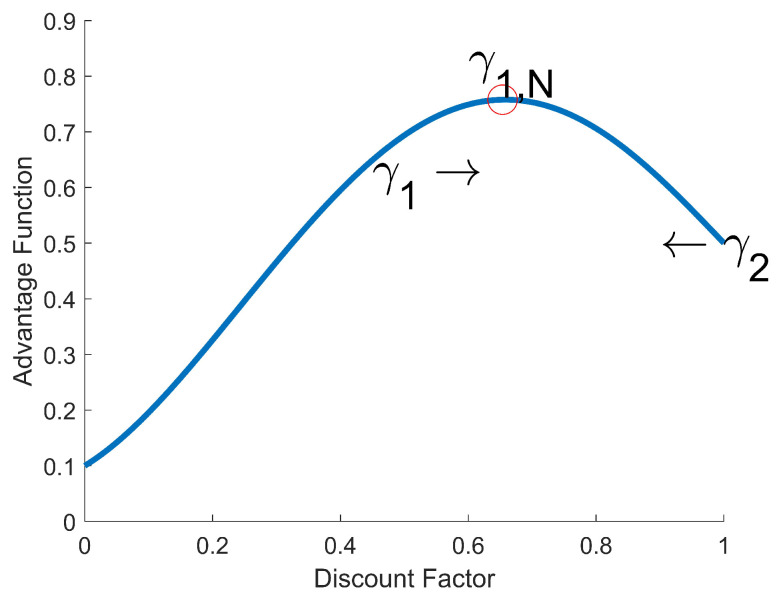
Example of discount factor adjustment using the adaptive discount factor.

In light of (15), the advantage function $A_{t}\left(\gamma_{1}\right)$ can be viewed as an ($t_{end}-1$)th-order polynomial in $\gamma_{1}$. Figure 5 shows an example of a graph for the advantage function. This means that $\gamma_{1}$ and $\gamma_{2}$ have to meet at a point where the maximum of the advantage function is achieved like the red circle in Figure 5.

Thanks to this, the proposed method can automatically find the maximum advantage function between the discount factor 0.5 and 0.99. A discount factor of less than 0.5 is ignored because it is too small to take a long-term reward sum into account. Since the return $\sum_{l=t}^{t_{end}}\gamma_{1,N}^{l-1}$ in (15) is proportional to the objective function of policy (5), the update size of the policy gradient can be larger when $\gamma_{1,k}$ is updated to $\gamma_{1,N}$. In summary, the return and discount factor are evaluated by the advantage function, the discount factor is determined according to that evaluation, and the return with a higher advantage function value is considered further in the training.

In the next sections, it is shown how the proposed adaptive rule can be implemented in both on-policy and off-policy reinforcement learning algorithms. This is necessary because the advantage function has to be computed differently in on-policy and off-policy reinforcement learning algorithms.

### 4.2. Adaptive Discount Factor in On-Policy RL

For the purpose of presenting how to apply the proposed method to an on-policy algorithm, PPO (Proximal Policy Optimization) is employed in this paper. For other on-policy algorithms, a similar procedure can be applied. PPO is a representative on-policy deep reinforcement learning algorithm that is a simplified method of the trust region method of TRPO (Trust Region Policy Optimization) [13,25].

The objective function for policy approximation in PPO is defined as (16), and the network parameter ϕ is tuned to maximize it.

(16) $$J^{CLIP}\left(\phi \right)=E\left[\min\left(\rho_{t}\left(\phi \right)\cdot A_{t},\;clip\left(\rho_{t}\left(\phi \right),1-\epsilon ,1+\epsilon \right)\cdot A_{t}\right)\right],$$

where $A_{t}$ is the advantage function and is defined after defining the value function approximation, and $\rho_{t}\left(\phi \right)$ denotes the policy conservation ratio and is defined as $\rho_{t}\left(\phi \right)=\frac{\pi_{\phi }\left(s_{t}|a_{t}\right)}{\pi_{\phi_{old}}\left(s_{t}|a_{t}\right)}$. In this way, when the policy $\pi_{\phi }\left(s_{t}|a_{t}\right)$ is learned, it is guaranteed not to degrade its performance compared with $\pi_{\phi_{old}}\left(s_{t}|a_{t}\right)$.

The adaptive discount factor algorithm (12) needs to compute two advantage (or value) functions, unlike the original PPO. To this end, two neural networks parameterized with $\psi_{1}$ and $\psi_{2}$ are trained in order to approximate each value function. The objective function for learning the state value functions $V_{\psi_{1}}$ and $V_{\psi_{2}}$ is given by

(17) $$J^{V}\left(\psi_{i=1,2}\right)=E\left[\frac{1}{2}{\left(r_{t}+\gamma_{i}V_{\psi_{i=1,2}}\left(s_{t+1}\right)-V_{\psi_{i=1,2}}\left(s_{t}\right)\right)}^{2}\right].$$

Based on the state value functions $V_{\psi_{1}}$ and $V_{\psi_{2}}$, the advantage function is computed as follows.

(18) $$A_{t}\left(\gamma_{i}\right)=r_{t+1}+\cdots +\gamma_{i}^{T-2-t}\cdot r_{T-1}+\gamma_{i}^{T-1-t}\cdot V_{\psi_{i}}\left(s_{T}\right)-V_{\psi_{i}}\left(s_{t}\right)$$

These advantage functions $A_{t}\left(\gamma_{i}\right),\;i=1,2$ are used in the proposed adaptive discount factor. During policy learning, $A_{t}\left(\gamma_{1}\right)$ is applied to the objective function (16) and is used in policy learning. PPO with the proposed adaptive discount factor is summarized in Algorithm A1.

### 4.3. Adaptive Discount Factor in Off-Policy RL

This section presents how to implement the proposed algorithm for off-policy deep reinforcement learning using SAC (Soft Actor-Critic). SAC is a model-free reinforcement learning algorithm suitable for continuous action tasks [16,26].

Similar to the previous section, two value functions parameterized by $\psi_{1}$ and $\psi_{2}$ are needed for the adaptive algorithm. The objective function for approximating the state value function of SAC is defined as follows:

(19) $$J^{V}\left(\psi_{i=1,2}\right)=\frac{1}{2}{\left(V_{\psi_{i}}\left(s_{t}\right)-E_{a_{t}}\left[Q_{\theta_{i}}\left(s_{t},a_{t}\right)-\alpha \log\pi_{\phi }\left(a_{t}|s_{t}\right)\right]\right)}^{2},$$

where $-\alpha \log\pi_{\phi }\left(a_{t}|s_{t}\right)$ is the entropy of the policy distribution, the constant α is a tuning parameter and determines the ratio of entropy, and  $Q_{\theta_{i}}$ is the action value function parameterized by $\theta_{1}$ and $\theta_{2}$. The following objective function is used to approximate the action value function.

(20) $$J^{Q}\left(\theta_{i=1,2}\right)=\frac{1}{2}{\left(Q_{\theta_{i}}\left(s_{t},a_{t}\right)-\left(r\left(s_{t},a_{t}\right)+\gamma_{i}V_{\psi_{i}}\left(s_{t+1}\right)\right)\right)}^{2}$$

The neural network to approximate the policy is trained using the following objective function:

(21) $$J^{\pi }\left(\phi \right)=\log\pi_{\phi }\left(a_{t}|s_{t}\right)-Q_{\theta_{1}}\left(s_{t},a_{t}\right)$$

where the right hand side is nothing but the difference between the distribution of the policy and the distribution of the action value function. Hence, the objective function (21) is the form of a Kullback–Leibler divergence between $\pi_{\phi }\left(a_{t}|s_{t}\right)$ and $Q_{\theta_{1}}\left(s_{t},a_{t}\right)$.

With these definitions in mind, in SAC, the advantage function for the adaptive algorithm can be defined as

(22) $$A_{t}\left(\gamma_{i=1,2}\right)=Q_{\theta_{i}}\left(s_{t},a_{t}\right)-\alpha \log\pi_{\phi }\left(a_{t}|s_{t}\right)-V_{\psi_{i}}\left(s_{t}\right).$$

Note that entropy is added to the definition of the advantage function (11). Since the state value function $V_{\psi_{i}}\left(s_{t}\right)$ approximates the reward sum using the entropy-augmented reward in SAC, it is also considered in the advantage function. The SAC with the proposed adaptive algorithm is summarized in Algorithm A2.

## 5. Experiment in the Environments with Uncertainty

In this section, applications of the proposed adaptive algorithm are shown to validate the performance. To test the performance of the on-policy and off-policy algorithms, Tetris game agent and motion planning for a robot manipulator are presented.

The adaptive discount factor algorithm is compared with fixed discount factors and the progressively increasing discount factor. The method of the progressively increasing discount factor is adopted from [15]. This increasing rule is described by

(23) $$\gamma_{1,k+1}=1-0.98\left(1-\gamma_{1,k}\right).$$

The discount factor $\gamma_{1,k}$ denotes a value of $\gamma_{1}$ at the *k*th training epoch. The initial and final values of $\gamma_{1,k}$ are set to 0.5 and 0.99, respectively. This increasing discount factor method shows better policy improvements compared with a constant discount factor [15].

### 5.1. Tetris

Tetris has been used a lot as a challenge in the field of artificial intelligence, and there have been efforts to solve it with various machine learning algorithms [27,28,29]. However, Tetris is known as an NP-hard problem, and it is a challenging problem with deep reinforcement learning [28]. Additionally, as Tetris is an environment with inherent uncertainty, it is not easy to estimate the maximum reward sum by approximating the value function [3]. Tetris is a game in which randomly given blocks, called tetrominoes, are stacked on the bottom, and if the blocks are filled without empty spaces, a score proportional to the number of filled rows is given. Tetris basically amounts to a continuing task, but if the player stacks up blocks without filling until the top, the game ends there. Thus, an overestimation of a reward for the time far away from the current time can mislead the game without realizing the uncertainty. For this, the proposed adaptive discount factor can be a suitable method for developing an RL-based game agent for Tetris.

Tetris is a discrete action task, and at every step you can move blocks left, right, down, or rotate $90^{\circ }$. There are two kinds of drop actions: soft and hard drops. The soft drop moves the block down by one space, and the hard drop, to the bottom.

In this paper, we redefine the agent’s action space as a compound action, like [30]. Compound action space A={0,1,2,⋯,35} is defined, which consists of move left/right (9), rotate a block (4), and a hard drop (1). The compound actions can speed up learning by limiting the agent’s meaningless actions. The Tetris game screen is 10×20 in this paper.

**Figure 6:**
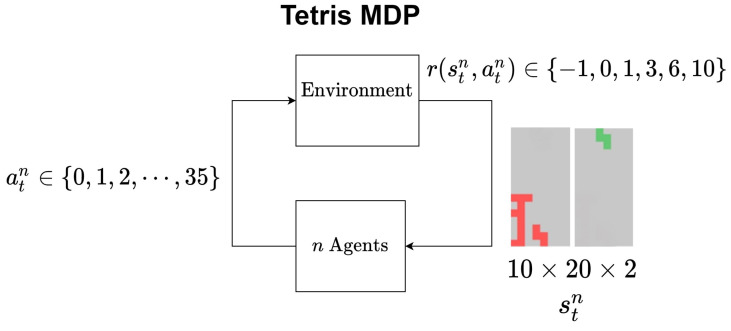
Schematic of MDP in the game of Tetris.

The state of MDP is composed of two images of the screen, i.e., 10×20×2. One is the image of the currently stacked blocks without the currently controllable block, and the other is the images of the controllable blocks without the currently stacked blocks. See Figure 6. The reward is determined based mainly on the number of lines cleared by the agent. Table 1 defines the reward.

Here, $n_{r}$ denotes the number of cleared lines by the current action, and *r*, the corresponding reward. −∞ in the second column in Table 1 describes that the blocks reach the top. In other words, the agent loses the game. Figure 6 depicts the structure of MDP of Tetris.

**Figure 7:**
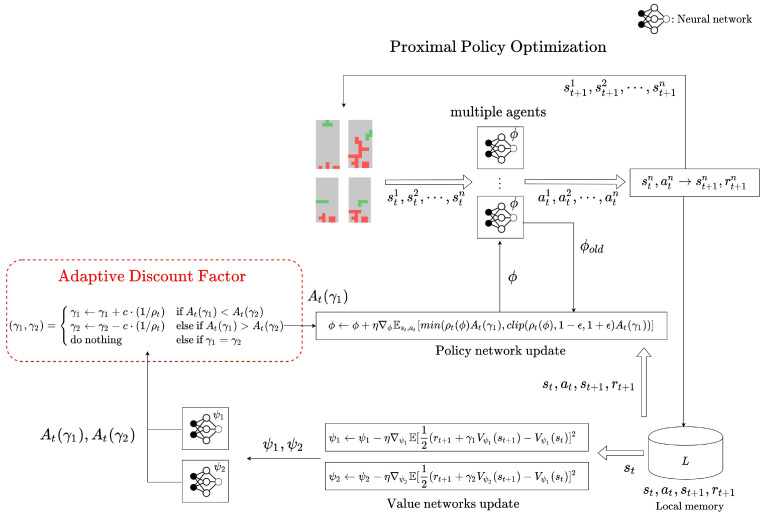
PPO with adaptive discount factor for the Tetris environment.

The PPO algorithm is implemented using a parallel agent method such as A3C (Asynchronous Advantage Actor-Critic) for efficient learning, and is applied to develop a Tetris game agent for fast performance improvement. The A3C algorithm samples data from multiple agents in parallel, and uses them for training. When the number of a specific agent is *n*, the data obtained from the *n*th agent in the time step *t* is $\left(s_{t}^{n},a_{t}^{n},r_{t}^{n},s_{t+1}^{n}\right)$. Each agent decides a policy through the same neural network with parameters ϕ, and synchronizes all neural networks whenever policy learning is performed. Figure 7 describes how the proposed adaptive discount factor is used to devise a PPO-based game agent for Tetris.

The performance of Tetris can be evaluated by the number of lines cleared by the agent during one episode. Learning performance with the adaptive discount factor is compared with the performance with fixed discount factors.

**Figure 8:**
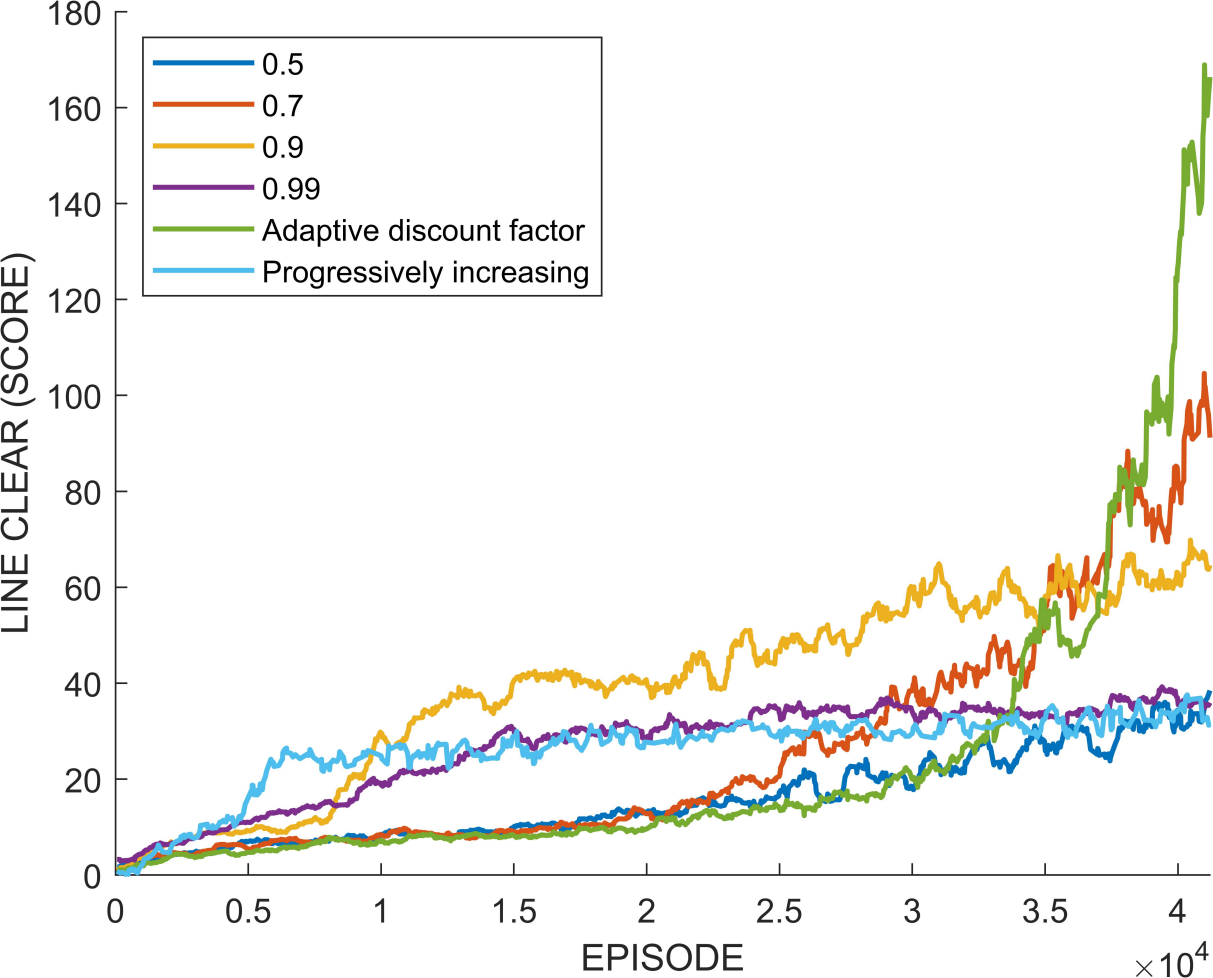
Performance comparison between fixed and adaptive discount factor in Tetris.

Figure 8 shows training performances by four different fixed discount factors, the proposed adaptive discount factor, and the progressively increasing discount factor. The discount factor of 0.99 is a commonly used value in deep reinforcement learning, and 0.5 is the low limit of the adaptive discount factor. The horizon axis of the graph indicates the number of episodes learned by the agent, and the vertical axis indicates the number of lines cleared by the agent for each episode. The highest discount factor of 0.99 shows a fast performance improvement at the beginning of learning, but the performance converges to a certain level as the episode progresses. The lowest discount factor of 0.5 shows the lowest performance compared to other discount factors. The discount factor of 0.9 shows a faster performance improvement than 0.99 at the beginning of training. It is confirmed that the discount factor of 0.7 can reach the highest performance, although the performance improvement is slow at the beginning of training. The performance of the progressively increasing discount factor was improved quickly at the beginning, but in the end, it converged to a similar performance by the fixed discount factor of 0.99. The performance of the agent trained using the adaptive discount factor is lower than those by the fixed discount factors at the beginning of training, but the final performance exceeds that of the agent using a discount factor of 0.7.

**Figure 9:**
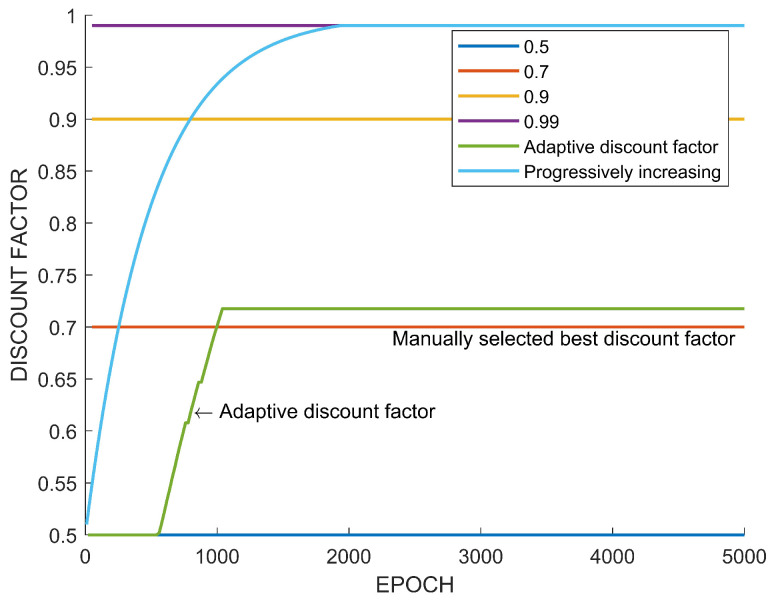
Adjustment of discount factor in Tetris.

Figure 9 shows the used fixed discount factors and the resulting adaptive discount factor and the increasing discount factor. In the case of the adaptive discount factor, adjustment is stopped when the discount factor reaches $\gamma_{1}=\gamma_{2}$. The adaptive discount factor is stopped at $\gamma_{1}=0.7214$. At the beginning of the adjustment, $\gamma_{1}$ does not change because $A_{t}\left(\gamma_{1}=0.5\right)>A_{t}\left(\gamma_{2}\right)$. However, when $A_{t}\left(\gamma_{1}=0.5\right)<A_{t}\left(\gamma_{2}=0.7214\right)$, $\gamma_{1}$ is increased, as is shown in Figure 9. Note that the adaptive discount factor stops adjusting at the value near 0.7, which results in a high performance among the fixed discount factors in Figure 8. Table 2 shows the comparison of the final performance of the fixed discount factors and the adaptive discount factor.

In Table 2, the scores in bold mean the highest maximum score or highest average score along the discount factors and algorithms. Also, the discount factors in bold are the values corresponding to the maximum score.

### 5.2. Motion Planning

**Figure 10:**
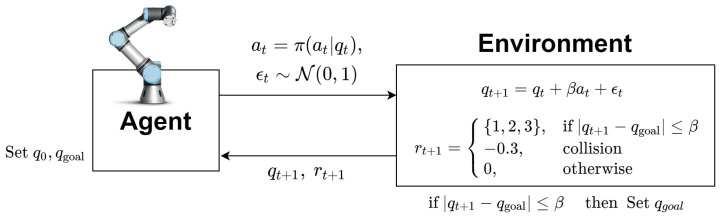
Schematic of MDP in robot manipulation task.

This section presents an application of the proposed adaptive discount factor to the path planning of the robot manipulator for the purpose of validating the proposed method for off-policy RL [31,32]. The joint value of the robot arm is expressed in the configuration space, and the current state representing the joint value of the robot arm at the current time step *t* is denoted by $q_{t}\in Q$ [33,34]. The action $a_{t}$ is the amount of change in the joint value, and the action space is given by A=(0,1). Since the action of the agent is continuous, the SAC algorithm is employed for motion planning. In general, although path planning problems are achieved via simulation, which means that there are no uncertainties in the problem, in order to consider real environments in this paper, two uncertainties are added to the path planning problem in the simulation. First, noise $\epsilon_{t}$ is added to the state evolution. In other words, the next state is determined by $q_{t+1}=q_{t}+\beta a_{t}+\epsilon_{t}$, where β is a constant, $a_{t}$ is the amount of change in the joint value, and the noise $\epsilon_{t}∼N\left(0,1\right)$. Second, a reward signal is transmitted probabilistically. In general, in the path planning task, the reward signal is given a positive value when the agent arrives at a goal point, and a negative value when it collides [35]. In this paper, when the agent arrives at the goal point, one of {1,2,3} is randomly transmitted. Therefore, the agent cannot easily approximate the reward sum at the current point. Additionally, since there is noise in the environment, the agent cannot easily determine whether to prioritize collision avoidance or to prioritize goal arrival, even if collision is considered. In addition, the problem of uncertainty is more pronounced in continuing the task. In the path planning problem, an  episodic task ends when the goal is reached, but for a continuing task, a new goal is given to the agent [36]. This aspect makes it difficult for the path planning environment to predict the distant future rewards, and an overestimation has to be prevented. Therefore, the proposed adaptive discount factor can handle this difficulty appropriately. Figure 10 describes the MDP for the path planning based on the reinforcement learning.

**Figure 11:**
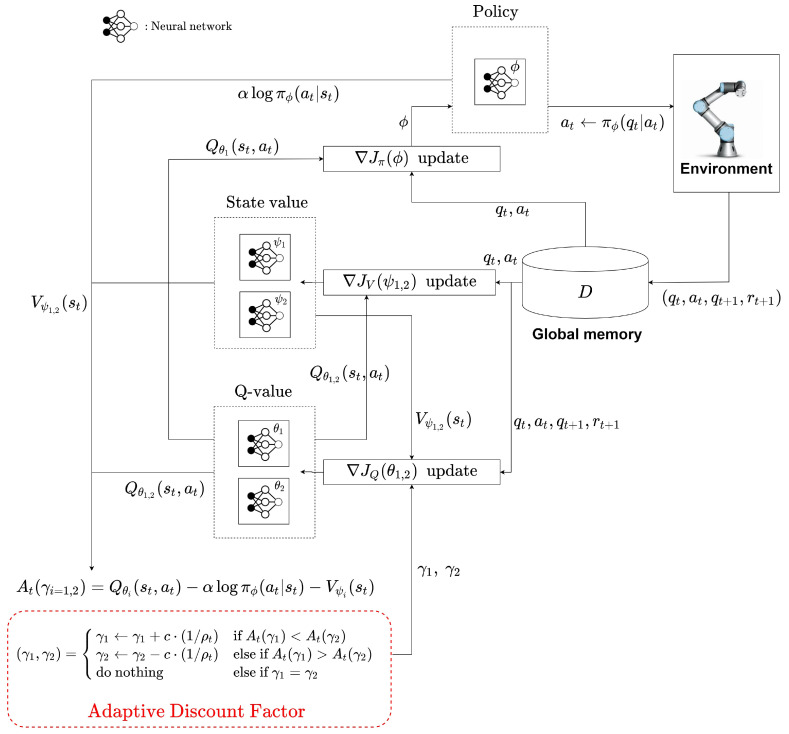
SAC with adaptive discount factor for robot manipulation environment.

In Figure 10, $q_{goal}$ represents the goal position given to the agent, and the inequality $|q_{t+1}-q_{goal}|\leq \beta$ is understood as the goal position is reached. Compared with PPO from the view point of applying the adaptive discount factor, the difference is in how to calculate the advantage function. The parameters of all neural networks are needed because SAC computes the advantage function based on Equation (22). Since SAC is an off-policy algorithm, it implements the experience replay memory *D*, randomly samples data during training, and applies it to each neural network training. Figure 11 describes how SAC with the adaptive discount factor is applied to the path planning of the robot manipulator.

For the validation of the performance of the path planning by the proposed method, two environments are considered. One is path planning in the form of an episodic task in which an episode terminates when the agent reaches the goal. The other is in the form of a continuing task in which a goal is given again when the agent arrives at the previous goal, and this is repeated until the agent collides. As explained before, the effect of the environmental uncertainty is more critical in the continuing task. In the episodic task, the episode ends when the agent reaches the goal, but in the continuing task, the next goal is given at a random location when the agent reaches the goal point. Therefore, in the case of the continuing task, it is not easy to estimate the expected reward sum. In this section, we test the learning performance of both the adaptive and fixed discount factors. In the path planning, the performance can be evaluated as the success ratio of the agent’s arrival at the goal point, that is, the success ratio of path creation. This success ratio is defined as the ratio of reaching the goal point in the last 10 episodes during training.

**Figure 12:**
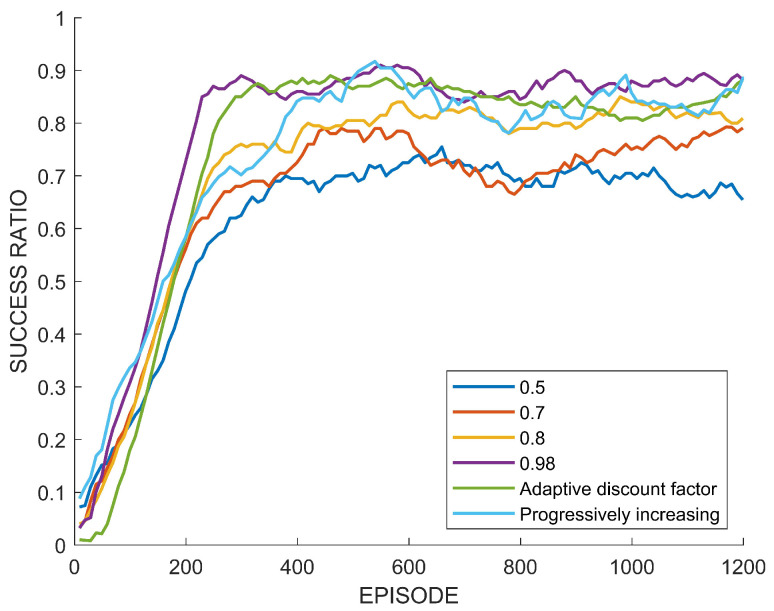
Success rate of path planning in the episodic task.

Figure 12 shows the learning performance of the fixed discount factors 0.5,0.7,0.8, and 0.98, the adaptive discount factor, and the increasing discount factor in the episodic task. The horizontal axis is the number of episodes, and the vertical axis is the success ratio of path generation. The lowest discount factor of 0.5 results in the lowest success rate. As the discount factor is increased, the success ratio is also increased. When the adaptive discount factor is applied, it shows a somewhat low performance at the beginning, but as learning progresses, it can be seen that the performance is closest to the fixed discount factor of 0.98. The performance of the increasing discount factor method was also similar and reached a high performance. Since, in the episodic task, the uncertainty is low and the reward sum is easy to predict, the fixed discount factor of 0.98 shows a higher performance.

**Figure 13:**
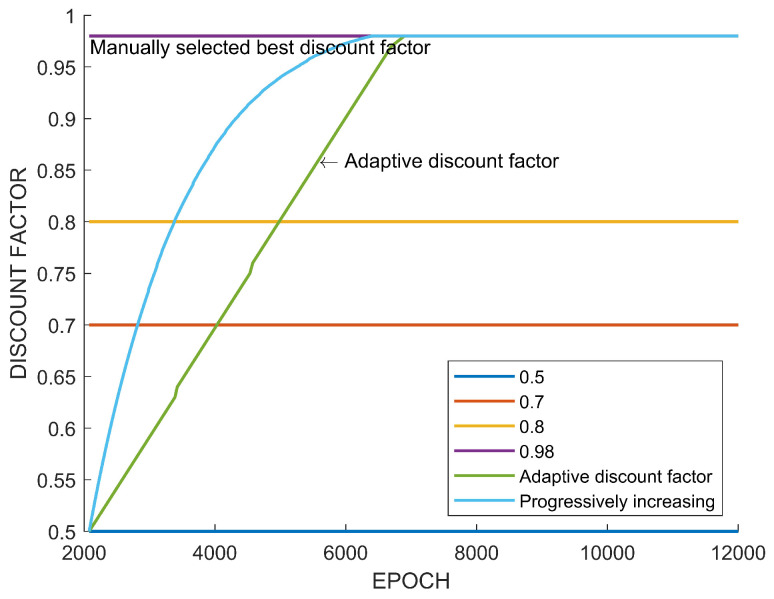
Adjustment of discount factor in episodic path planning task.

Figure 13 compares the evolution of the adaptive discount factor with other fixed discount factors in the episodic work. It can be seen that the adaptive discount factor converges close to the highest discount factor of 0.98 as the training goes on. A high discount factor is advantageous for learning for the path planning in episodic tasks, since a reward signal is given and the episode ends when the final goal is reached. Furthermore, since the path planning problem is generally regarded as a sparse reward problem, a high discount factor is helpful. Table 3 shows the success ratio of path creation according to each discount factor. In Table 3, the success ratio is the average of the past 10 test episodes, and if the path generation succeeds in all 10 times, it is 1.0. The values of the success rate in bold are the highest value among them and the discount factors in bold are the optimal values in the episodic environment.

In the following, the learning performance of the adaptive discount factor, increasing discount factor, and the fixed discount factor in the path planning is compared in a continuing task environment. When the agent reaches the current goal point, a new goal point is given, and this is repeated. In other words, in a continuing task, the episode continues until the agent collides. For this case, the learning performance is evaluated by the number of paths generated by the agent before collision.

**Figure 14:**
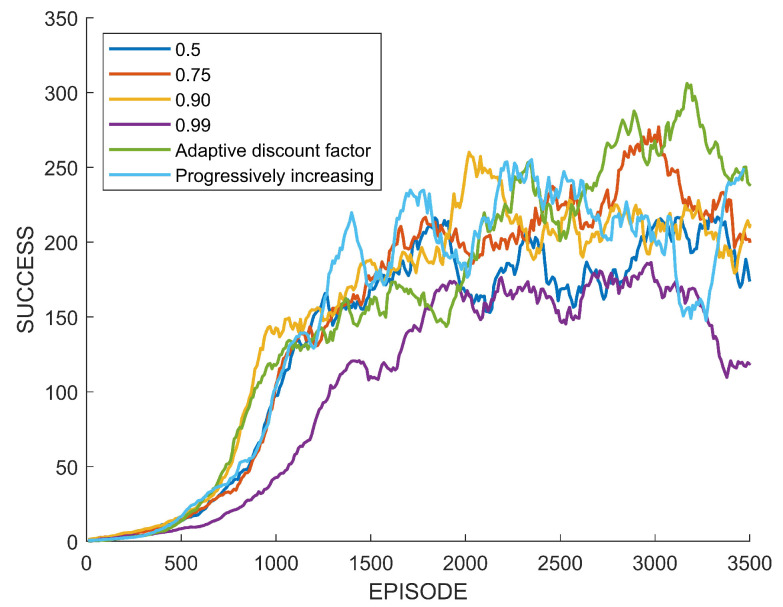
Perforamance of path planning in continuing task.

Figure 14 shows the learning performance of the fixed discount factors of 0.5, 0.75, 0.85, and 0.99, increasing the discount factor and the adaptive discount factor for the path planning in the continuous episode setting. Three observations can be made. First, among the fixed discount factors, the resulting performance is not monotone. Namely, it does not hold that the higher the discount factor is, the better the performance that is generated. Second, except for a discount factor of 0.99, all of the other cases including the adaptive discount factor and the increasing discount factor show similar performances during the transient time, and a performance of 0.99 was not high, even at the end of training. Third, the adaptive discount factor shows the best performance in the end.

**Figure 15:**
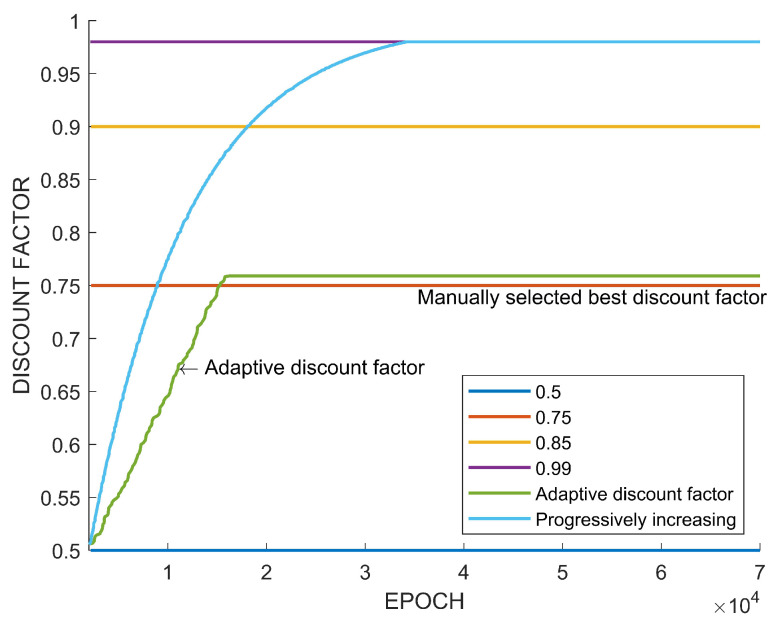
Adjustment of discount factor in continuing path planning task.

Figure 15 shows the evolution of the adaptive discount factor and the fixed discount factors. Note that a fixed discount factor of 0.75 results in the best performance and the adaptive discount factor converges to a value of near 0.75. Table 4 summarizes the performance according to each discount factor. The numbers of created paths in bold mean the highest maximum or the highest average number of paths according to algorithms and discount factors. Also, the discount factors in bold correspond to the highest performance.

Hence, it is verified that the proposed adaptive discount factor leads to a good performance, although there are inherent uncertainties in the environment, and the reward is sparse.

In view of these case studies, it is confirmed that the deep RL with the proposed adaptive discount factor results in a comparable learning performance with that by the best and fixed discount factor that has to be determined by exhaustive simulations.

### 5.3. Analysis and Discussion

In view of the previous case study, it is confirmed that the adaptive discount factor is indeed effective. In many existing results on reinforcement learning, a large discount factor is usually used, such as 0.99. However, as seen from the case study, the high discount factor does not necessarily always guarantee a high performance. In the previous learning experiment, when a discount factor was randomly selected among values between 0.5 and 0.99 and training was performed, there was a case in which a higher performance was obtained with a value of lower than 0.99, as shown in Figure 8 and Figure 14. On the other hand, Figure 12 showed the highest performance, with 0.99. The difference between these tasks is the existence of an uncertain risk or an unpredictable negative reward among distant future rewards. In the case of Tetris, the agent might obtain negative rewards according to future mistakes, but it is impossible to predict this because blocks are randomly determined. In the case of path planning, it is impossible to predict the failure and collision of path generation when generating a path to a new randomly generated goal point. These correspond to the example in Figure 3. On the other hand, in the case of the path planning problem that is an episodic task, it is relatively easy to predict the success or failure of path creation for a fixed goal point. This case only matches Figure 4, which is a sparse reward environment. Therefore, the discount factor of 0.99 showed the highest performance in this task. The adaptive discount factor algorithm closely finds the discount factor that can give the best performance in all cases. This saves the effort in finding a suitable discount factor. Additionally, as shown in Figure 9, Figure 13 and Figure 15, the adjustment of the discount factor is quickly terminated at the beginning of training. Since the adjusted discount factor is fixed and learning proceeds with the fixed value, it is not computational expensive. Another discount factor adjustment algorithm, called progressively increasing discount factor [15], gradually increases the discount rate to 0.99. It has been suggested that this method can achieve a higher performance than a fixed discount factor. However, the proposed adaptive discount factor outperforms in an environment requiring a low discount factor. This also suggests that a somewhat lower discount factor may be appropriate, depending on the environment.

## 6. Conclusions

This paper proposed an adaptive discount factor for the environment with uncertainty. The discount factor has to be decided differently, according to the distribution of rewards given in the environment. A commonly used high discount factor places a high value on future rewards. However, depending on the environment, it can be risky to consider the distant future rewards too much. In this case, a high discount factor can impair the performance of the agent. In such an environment, a low discount factor can be an effective countermeasure. Generally, it is difficult to know what the appropriate discount factor is before training. Therefore, in this paper, we proposed an adaptive discount factor algorithm that can find a proper value of the discount factor automatically at the beginning of learning. The algorithm adjusts the discount factor according to the advantage function during training, such that the reinforcement learning agent shows good performance in the end. It is shown that the adaptive discount factor converges to the discount factor that is manually found via exhaustive search, and that leads to the best performance. Using Tetris and the path planning problem, we found that, depending on the environment, a slightly lower discount factor could yield a higher performance than a higher discount factor. Tetris has difficulty in predicting distant future rewards because of the next block that appears randomly. For the path planning problem, a high discount factor was suitable for an episodic task, but a low discount factor showed a high performance in a continuing task in which the goal point was randomly given. It is very cumbersome to find this from multiple test training. The proposed algorithm finds the discount factor by comparing the advantage functions created by different neural networks. In addition, in order to minimize the computational cost, it is performed quickly to search for the optimal discount factor at the beginning of learning. The proposed adaptive discount factor results consistently in a good performance for various environments.

Future research includes how to enhance the performance of the value estimation when there is a nontrivial gap between the model and the real environment. In this paper, the algorithm is applied in response to the simulation problem in which uncertainty exists. However, in real environments, more unpredictable situations may arise. Therefore, we would like to consider ways to adjust the trade-off between uncertainty and the value of rewards in real environments.

## Figures and Tables

**Table 1:** Number of cleared lines and reward.

$n_{r}$	−∞	0	1	2	3	4
*r*	−1	0	1	3	6	10

**Table 2:** Score comparison between fixed and adaptive discount factor in Tetris.

	Maximum Score	Average Score	$\gamma_{1}$
Adaptive discount factor	**318**	**139.28**	**0.7214**
Increasing discount factor	37	29.94	0.99
Fixed discount factor 1	49	25.98	0.99
Fixed discount factor 2	115	65.03	0.9
Fixed discount factor 3	**179**	**97.94**	**0.7**
Fixed discount factor 4	61	35.87	0.5

**Table 3:** Comparison of success rate in episodic path planning task.

	Success Rate	$\gamma_{1}$
Adaptive discount factor	0.8846	**0.98**
Increasing discount factor	**0.8915**	**0.98**
Fixed discount factor 1	**0.8884**	**0.98**
Fixed discount factor 2	0.8222	0.8
Fixed discount factor 3	0.7833	0.7
Fixed discount factor 4	0.6588	0.5

**Table 4:** Comparison of performance in continuing path planning task.

	Maximum	Averages	$\gamma_{1}$
Adaptive discount factor	**464**	**287.77**	**0.7591**
Increasing discount factor	409	194.74	0.99
Fixed discount factor 1	256	155.76	0.99
Fixed discount factor 2	351	212.87	0.90
Fixed discount factor 3	**421**	**231.86**	**0.75**
Fixed discount factor 4	388	189.09	0.5

## Data Availability

Not applicable.

## Appendix A

Algorithms A1 and A2 show the adaptive discount factor algorithms applied to PPO and SAC. In the algorithms, the advantage function is calculated using two neural networks, respectively, and the two discount factors $\gamma_{1}$ and $\gamma_{2}$ are evaluated according to the advantage functions. $\gamma_{1}$ is adjusted in the direction where the advantage function becomes larger, and when the two discount factors are equal, the adjustment is terminated.

**Algorithm A1:** Adaptive Discount Factor in PPO
1: Initialize the discount factors : $\gamma_{1}=0.5,\;\;\gamma_{2}=0.99$ 2: Initialize the network parameters : $\phi ,\;\psi_{1},\;\psi_{2}$ 3: **while** Training *N* epoch **do** 4: Samples *M* data from MDP 5: $G_{t}\left(\gamma_{i}\right)=r_{t+1}+\cdots +\gamma_{i}^{T-2-t}\cdot r_{T-1}+\gamma_{i}^{T-1-t}\cdot V_{\psi_{i}}\left(s_{T}\right)$ 6: $A_{t}\left(\gamma_{i}\right)=G_{t}\left(\gamma_{i}\right)-V_{\psi_{i}}\left(s_{t}\right)$ 7: **if** $\frac{1}{M}\sum^{M}A_{t}\left(\gamma_{1}\right)<\frac{1}{M}\sum^{M}A_{t}\left(\gamma_{2}\right)$ **then** 8: $\gamma_{1}\leftarrow \gamma_{1}+c\cdot \left(1/\sigma_{t}\right)$ 9: **else if** $\frac{1}{M}\sum^{M}A_{t}\left(\gamma_{1}\right)>\frac{1}{M}\sum^{M}A_{t}\left(\gamma_{2}\right)$ **then** 10: $\gamma_{2}\leftarrow \gamma_{2}-c\cdot \left(1/\sigma_{t}\right)$ 11: **else if** $\gamma_{1}=\gamma_{2}$ **then** 12: do nothing 13: **end if** 14: train policy parameter ϕ with $\gamma_{1}$ 15: $\phi \leftarrow argmax_{\phi }E\left[\min\left(\rho_{t}\left(\phi \right)\cdot A_{t}\left(\gamma_{1}\right),\;clip\left(\rho_{t}\left(\phi \right),1-\epsilon ,1+\epsilon \right)\cdot A_{t}\left(\gamma_{1}\right)\right)\right]$ 16: **end while**

**Algorithm A2:** Adaptive Discount Factor in SAC
1: Initialize the discount factors : $\gamma_{1}=0.5,\;\;\gamma_{2}=0.99$ 2: Initialize the network parameters : $\phi ,\;\psi_{1},\;\psi_{2},\;\theta$ 3: **while** Training *N* epoch **do** 4: Samples *M* data from Experience Replay Memory 5: $A_{t}\left(\gamma_{i=1,2}\right)=Q_{\theta_{i}}\left(s_{t},a_{t}\right)-\alpha \log\pi_{\phi }\left(a_{t}|s_{t}\right)-V_{\psi_{i}}\left(s_{t}\right)$ 6: **if** $\frac{1}{M}\sum^{M}A_{t}\left(\gamma_{1}\right)<\frac{1}{M}\sum^{M}A_{t}\left(\gamma_{2}\right)$ **then** 7: $\gamma_{1}\leftarrow \gamma_{1}+c\cdot \left(1/\sigma_{t}\right)$ 8: **else if** $\frac{1}{M}\sum^{M}A_{t}\left(\gamma_{1}\right)>\frac{1}{M}\sum^{M}A_{t}\left(\gamma_{2}\right)$ **then** 9: $\gamma_{2}\leftarrow \gamma_{2}-c\cdot \left(1/\sigma_{t}\right)$ 10: **else if** $\gamma_{1}=\gamma_{2}$ **then** 11: do nothing 12: **end if** 13: train policy parameter ϕ with $\gamma_{1}$ 14: $\theta_{i=1,2}\leftarrow argmin_{\theta_{i}}E\left[\frac{1}{2}{\left(Q_{\theta_{i}}\left(s_{t},a_{t}\right)-\left(r\left(s_{t},a_{t}\right)+\gamma_{i}V_{\psi_{i}}\left(s_{t+1}\right)\right)\right)}^{2}\right]$ 15: $\phi \leftarrow argmin_{\phi }E\left[\log\pi_{\phi }\left(a_{t}|s_{t}\right)-Q_{\theta_{1}}\left(s_{t},a_{t}\right)\right]$ 16: **end while**
